# Commencement

**Published:** 1877-03

**Authors:** 


### COMMENCEMENT.

The thirty-first annual commencement of the Ohio College of Dental Surgery was held on the evening of March 7, in the lecture room of the college, in the presence of a large assemblage of interested spectators.

The exercises were introduced by reading the scriptures and prayer by Rev. J. Chester. When the president of the board of trustees, Dr. James Taylor, after a short address, conferred the degree of Doctor of Dental Surgery on the following gentlemen, viz:

A. L. Brown, Ohio; J. B. Wirt, Ky.; Jas. S. McCampbell, Ohio; Thos. H. N. Smith, Ky,; J. B. Holderfield Ohio; W. J. Walker, Ky.

Immediately following this was the annual address, by Dr. E. J. Waye, of Sandusky, O., (which will appear in the next number of the REGISTER.) Then a response on behalf of the graduating class by A. L. Brown.

